# Supplementary material for: miR-410 Is a Key Regulator of Epithelial-to-Mesenchymal Transition with Biphasic Role in Prostate Cancer
Source: Cancers (Basel). 2023 Dec 21;16(1):48. doi: 10.3390/cancers16010048 (PMC10777946; doi:10.3390/cancers16010048)
Supplement: Supplementary file 1 [file cancers-16-00048-s001.zip › cancers-2695577-supplementary.pdf]

Supplemental figure legends:

Figure S1 miR-410 expression is downregulated upon treatment with dihydrotestosterone

LNCaP cells were treated with indicated concentration of DHT followed by real time PCR analyses of miR-410 expression. RNU48 was used as an endogenous control.

Figure S2 GSEA analyses of miR-410 target genes in PC3 cells (related to Figure 6C)

Enrichment scores for prominent pathways altered by miR-410 in PC3 cells are shown.

Figure S3 GSEA analyses of miR-410 target genes in C42B cells (related to Figure 6D)

Enrichment scores for prominent pathways altered by miR-410 in C42B cells are depicted. GSEA analyses was conducted on microarray data generated from C42B cells transfected with miR-CON/ miR-410.

Figure S4 Original Western Blot figures(related to Figures 5 and 7)
Blots showing the relative expression of indicated proteins in PC3, C42B and LNCaP cells.

Figure S1. miR-410 expression is downregulated upon treatment with dihydrotestosterone.

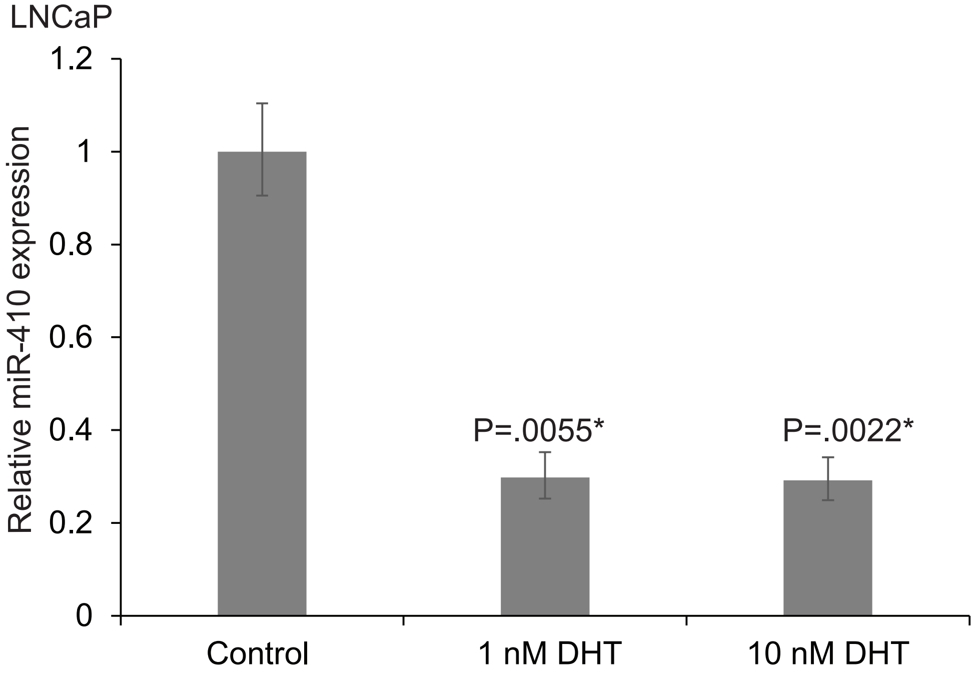

Figure S2. GSEA analyses of miR-410 target genes in PC3 cells.

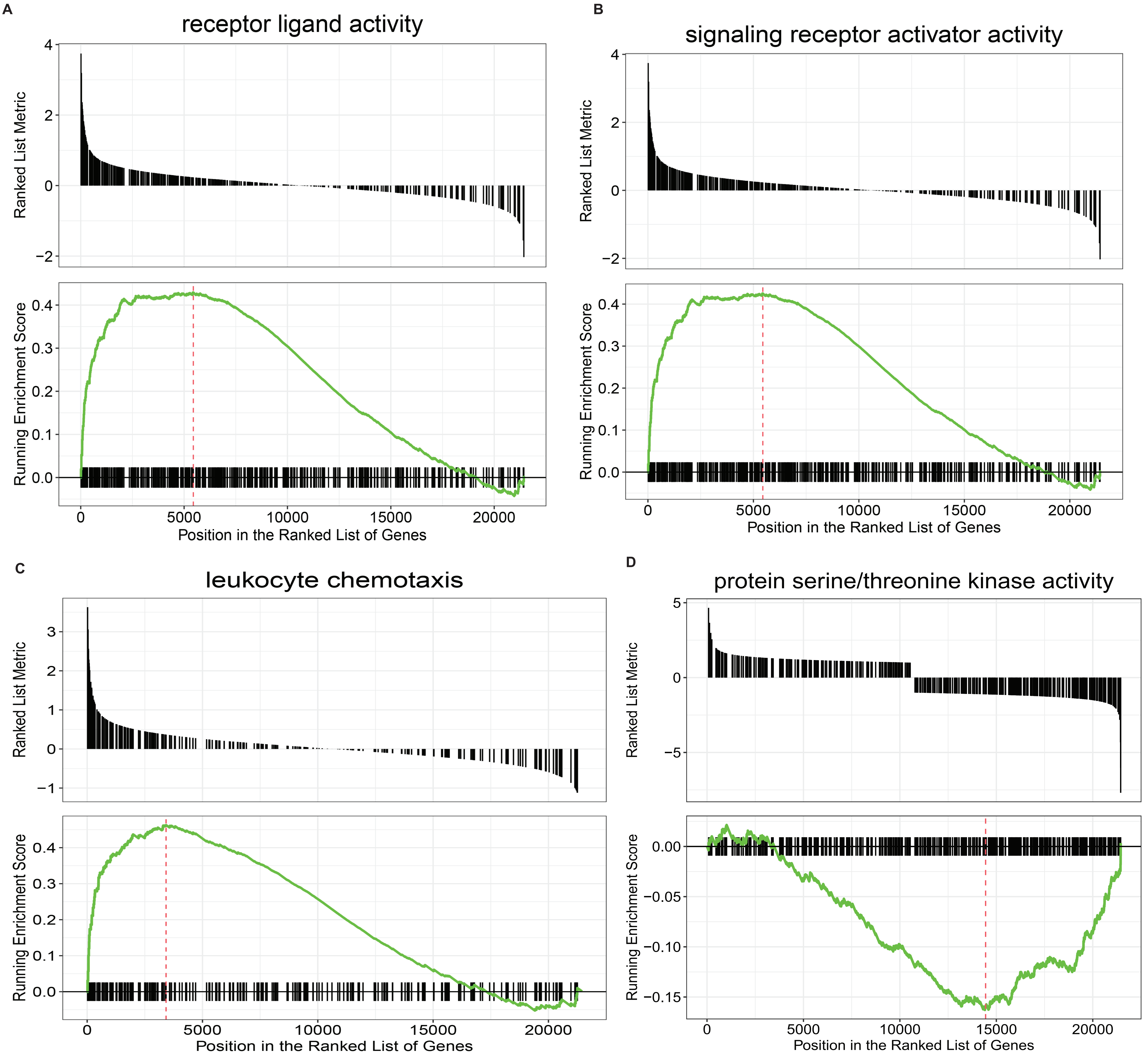

Figure S3 .GSEA analyses of miR-410 target genes in C42B cells.

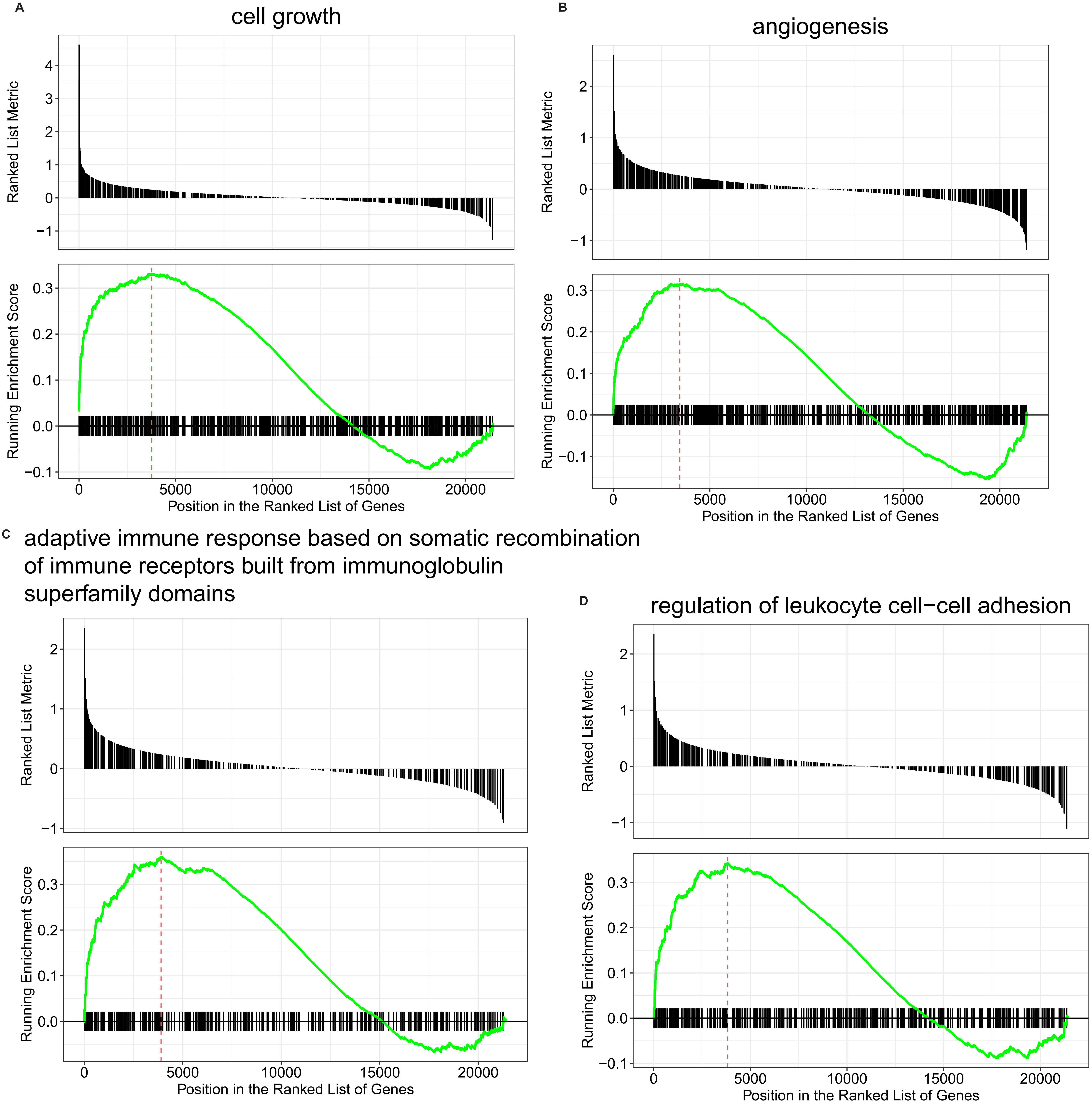

Figure S4. original Western Blot images.

E-cadherin and GAPDH for C42B miR-CON and C42B miR-410 as shown in Figure 5E.
GAPDH for PC3 miR-CON and PC3 miR-410 blot in PAN Akt blot as shown in Figure 7C
GAPDH for C42B miR-CON and C42B miR-410 in TGF-Beta blot as shown in Figure 7C

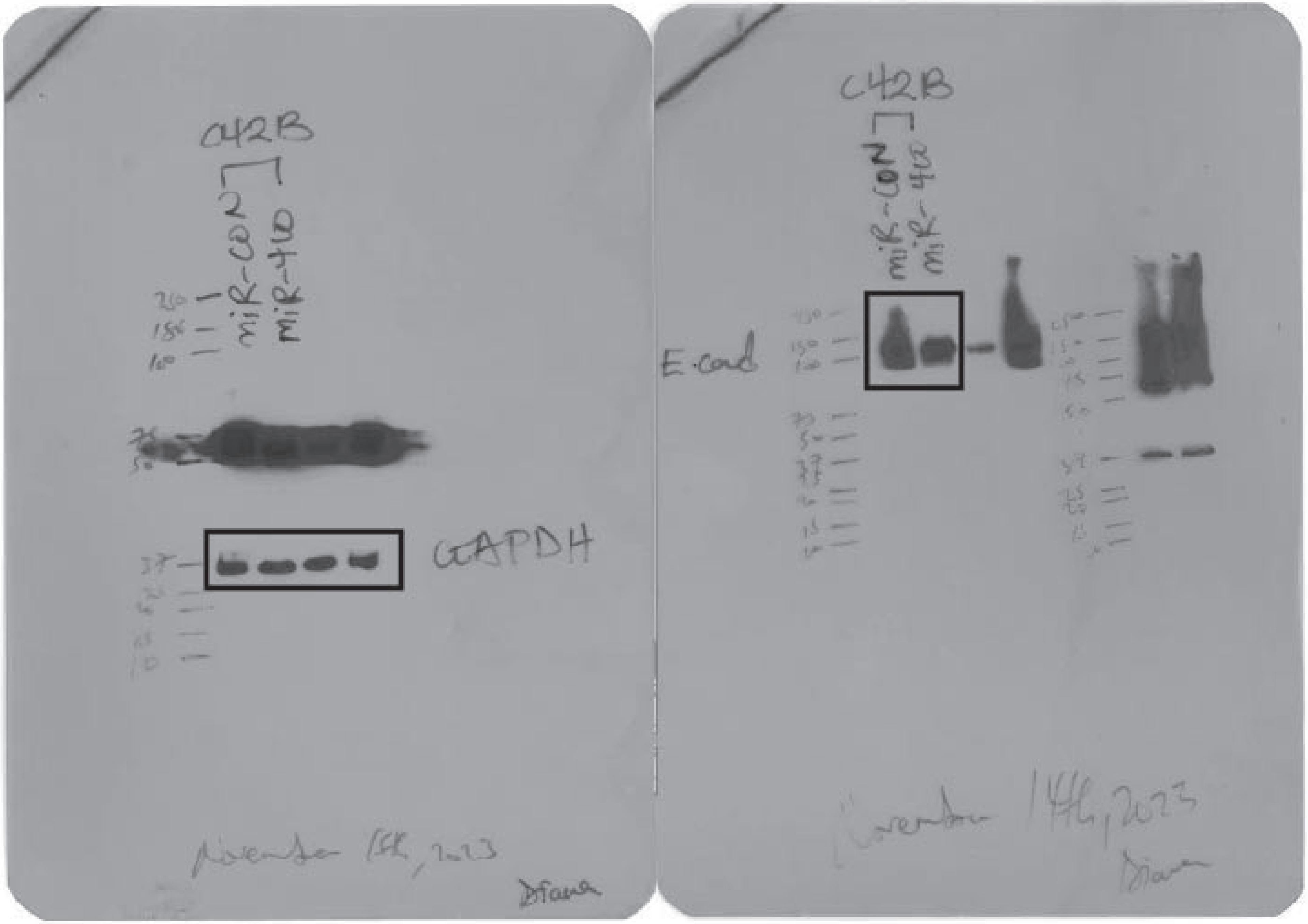

MMP10, SNAIL and GAPDH for C42B miR-CON, C42B miR-410, PC3 miR-CON, PC3 miR-410 and LNCaP miR-CON, and LNCaP miR-410 as shown in Figure 5C and Figure 5F

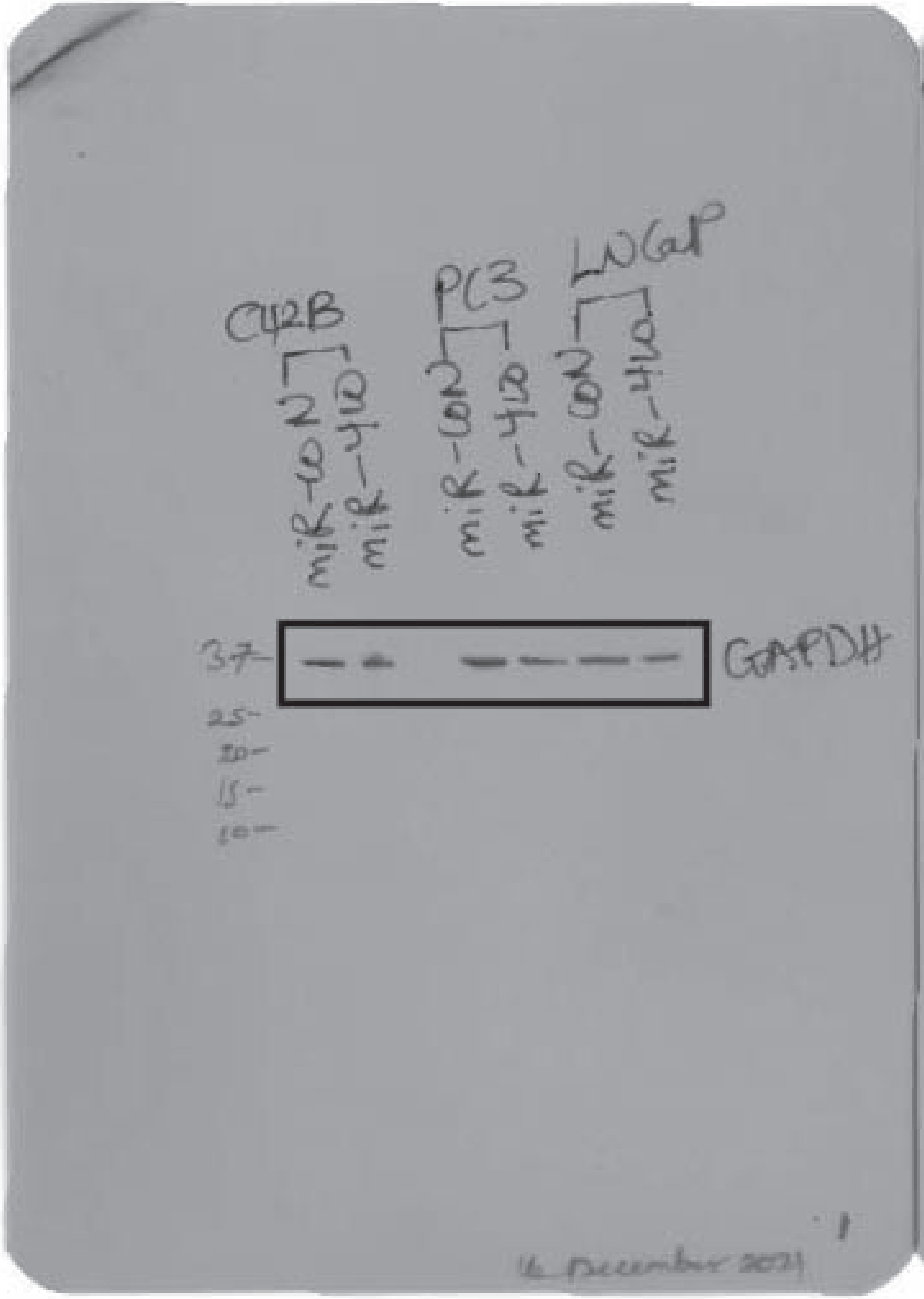

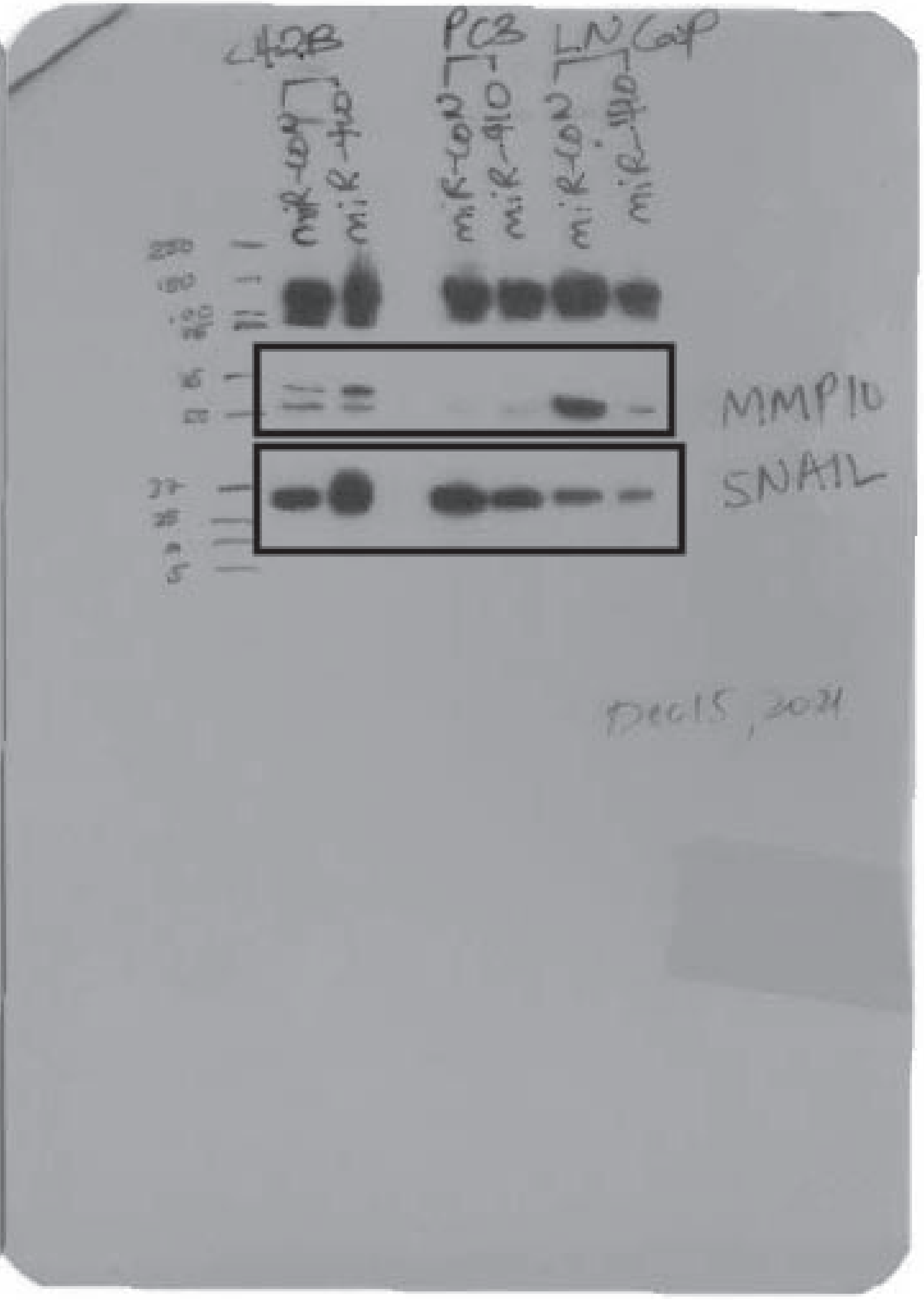

N-cadherin and GAPDH for LNCaP miR-CON and LNCaP miR-410 as shown in Figure 5A

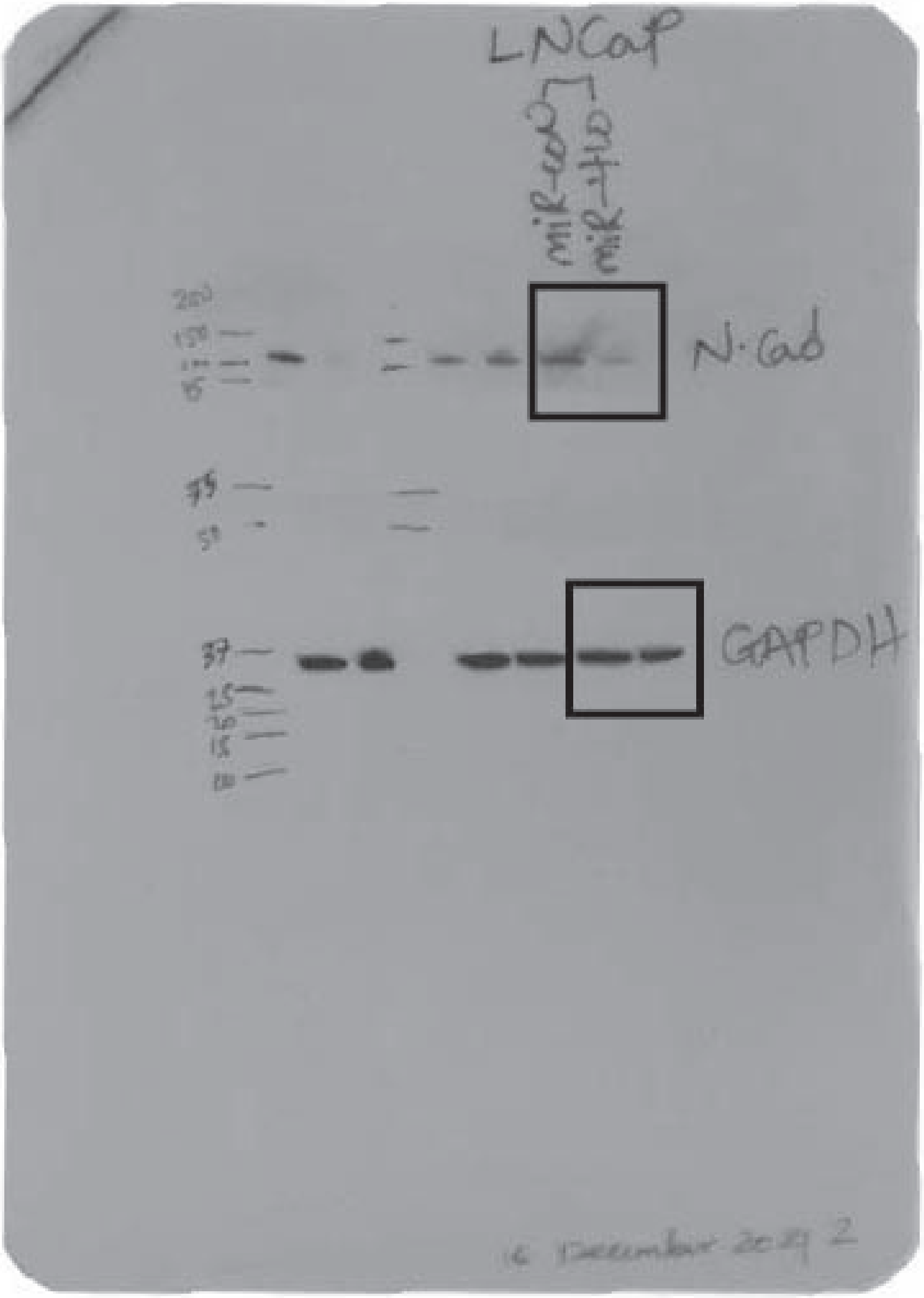

E-cadherin and GAPDH for LNCaP miR-CON and LNCaP miR-410 as shown in Figure 5A

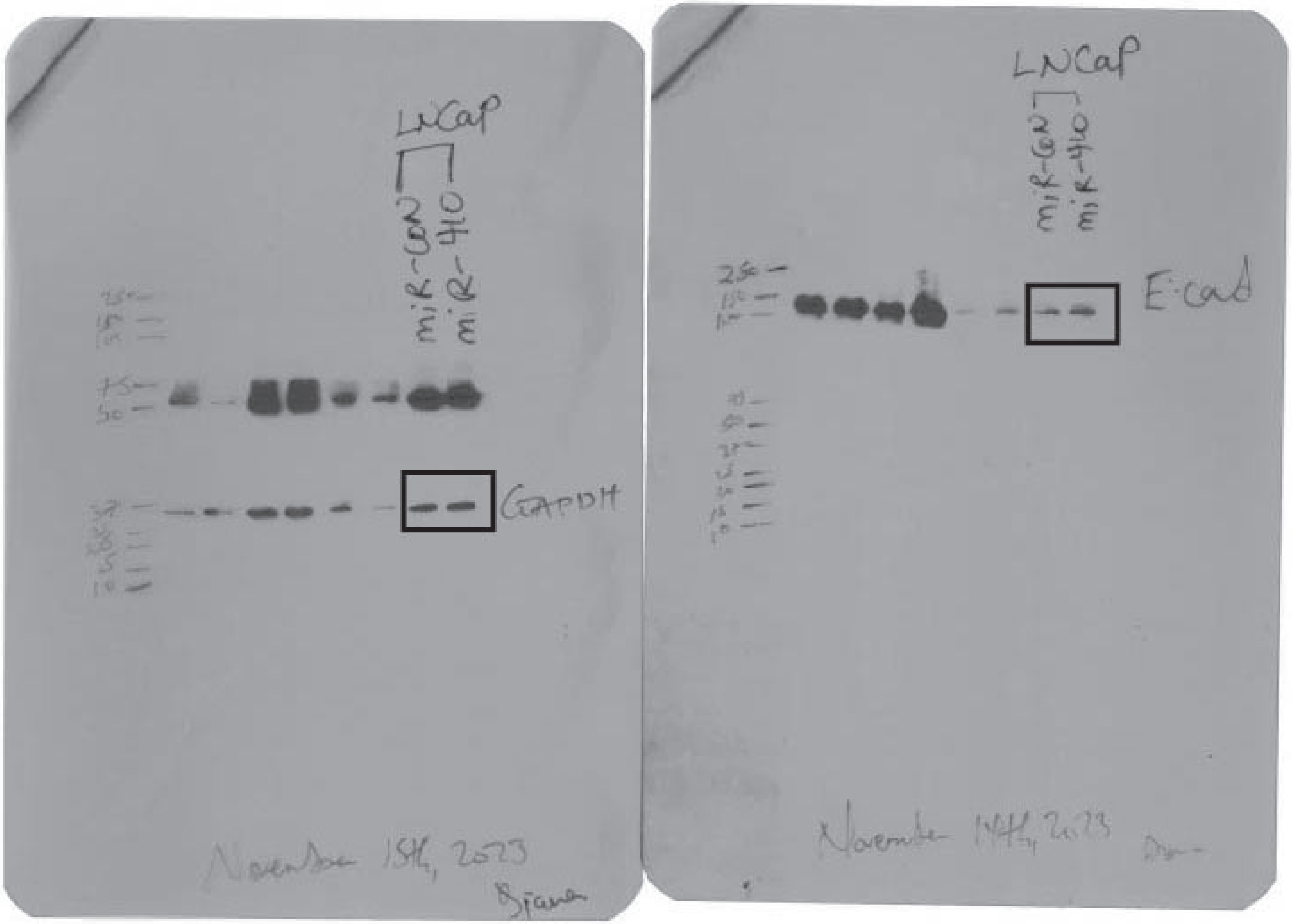

E-cadherin and GAPDH for PC3 miR-CON and PC3 miR-410 as shown in Figure 5E

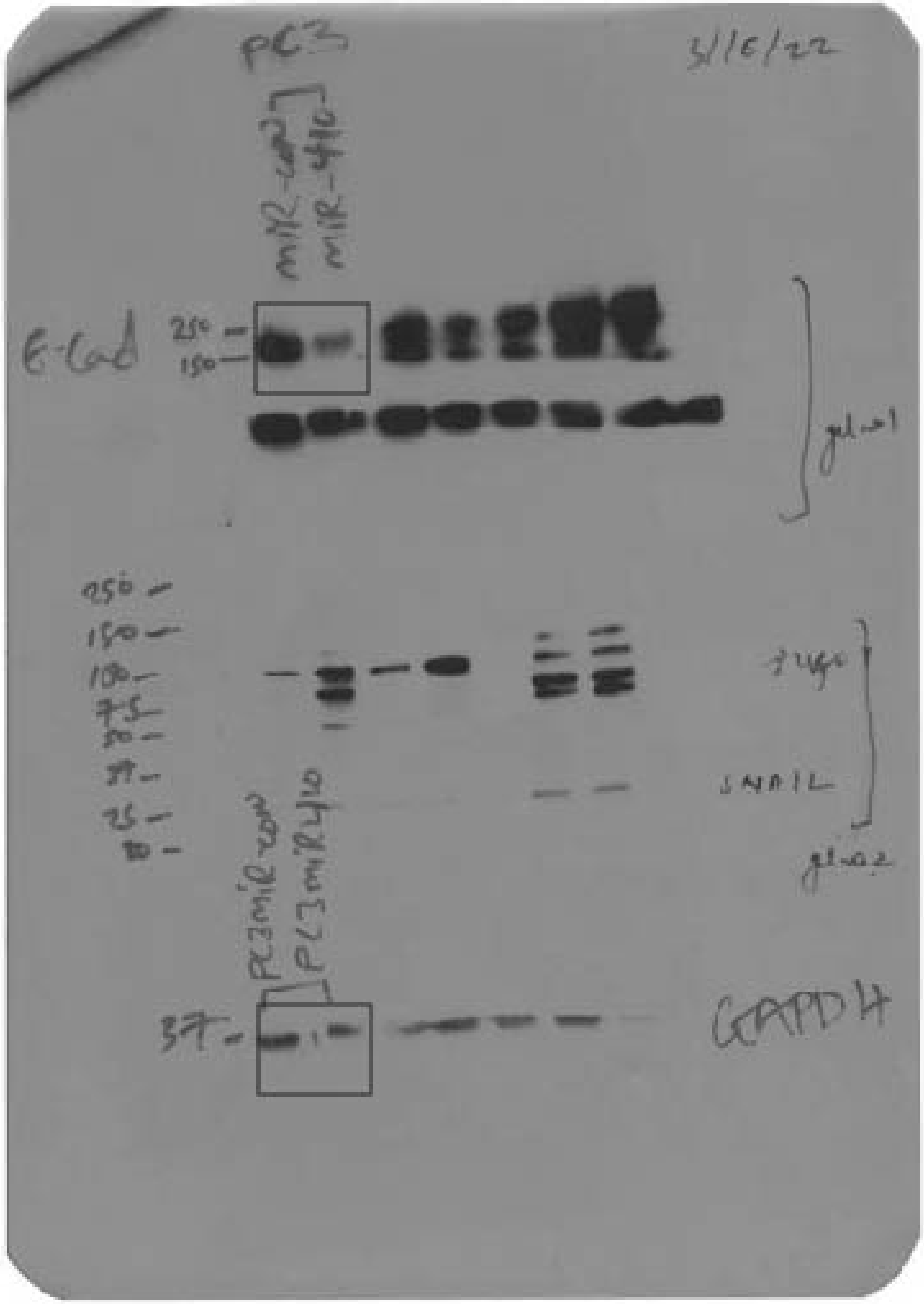

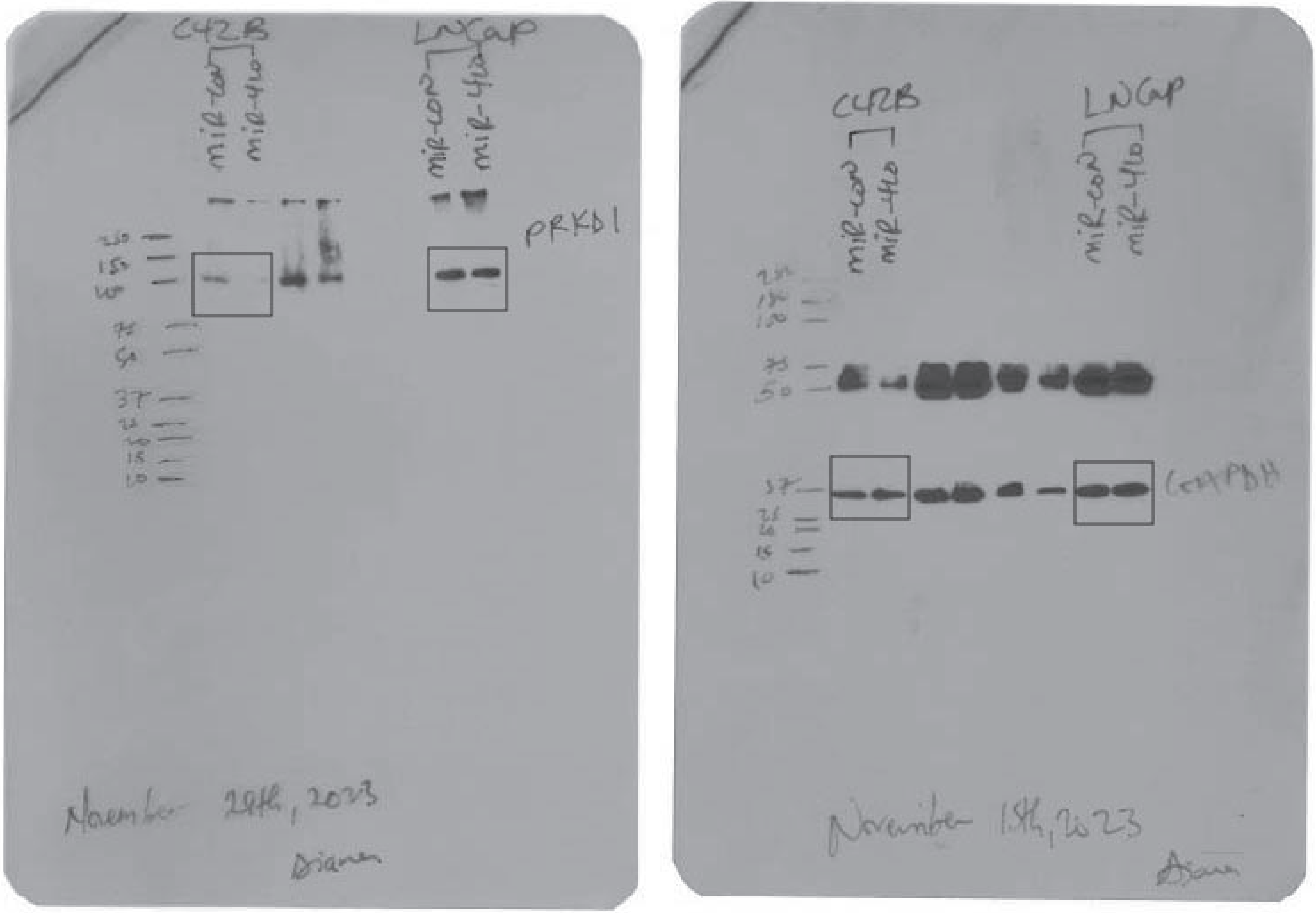

PRKD1 and GAPDH for C42B miR-CON, C42B miR-410, LNCaP miR-CON and LNCaP miR-410 shown in Figure 7B

AKT1 for PC3 miR-COn, PC3 miR-410, C42B miR-CON and C42B miR-410 as shown in Figure 7C

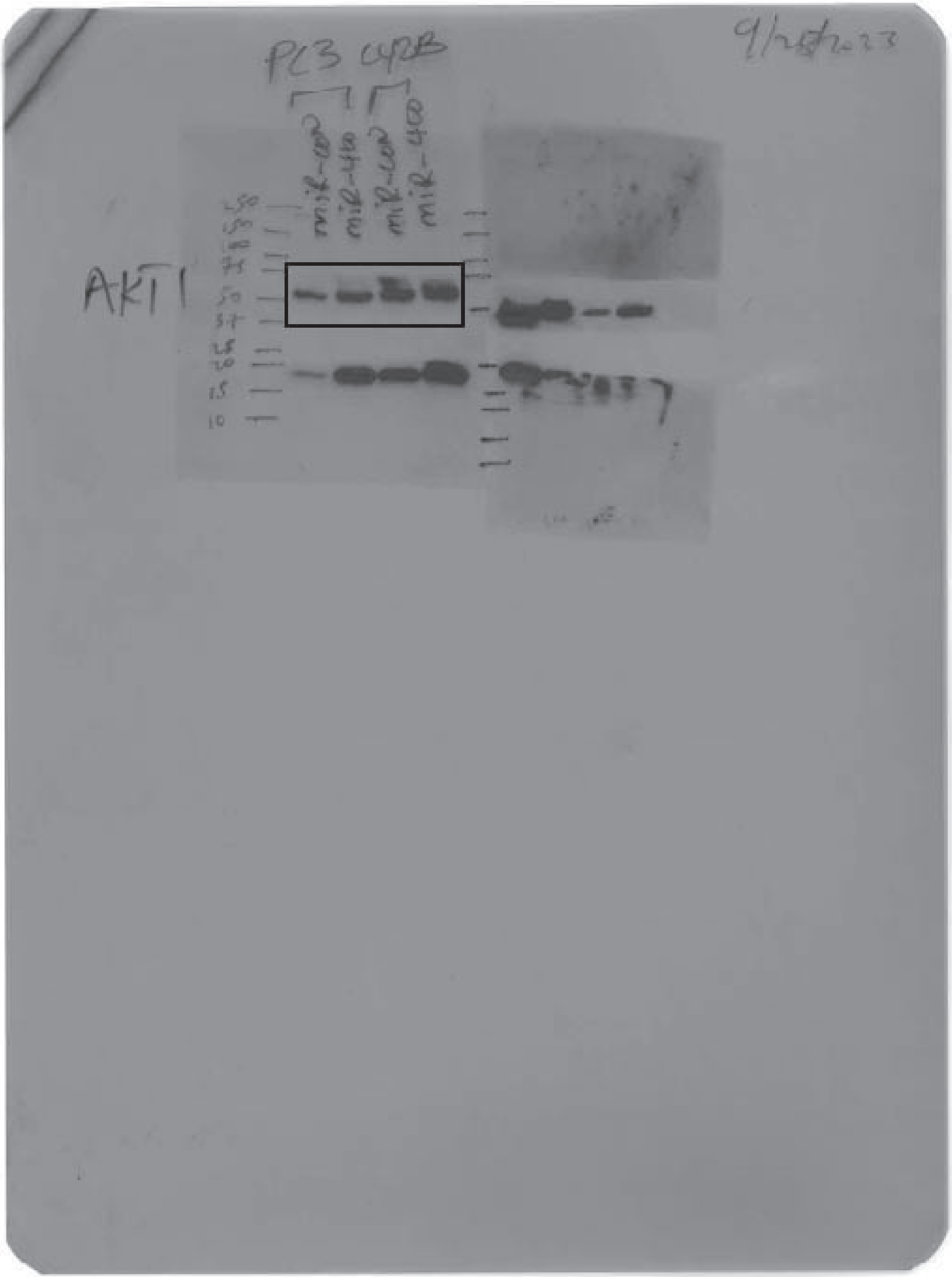

GAPDH for PC3 miR-CON, PC3 miR-410, C42B miR-CON and C42B miR-410 in AKT1 blot as shown in Figure 7C

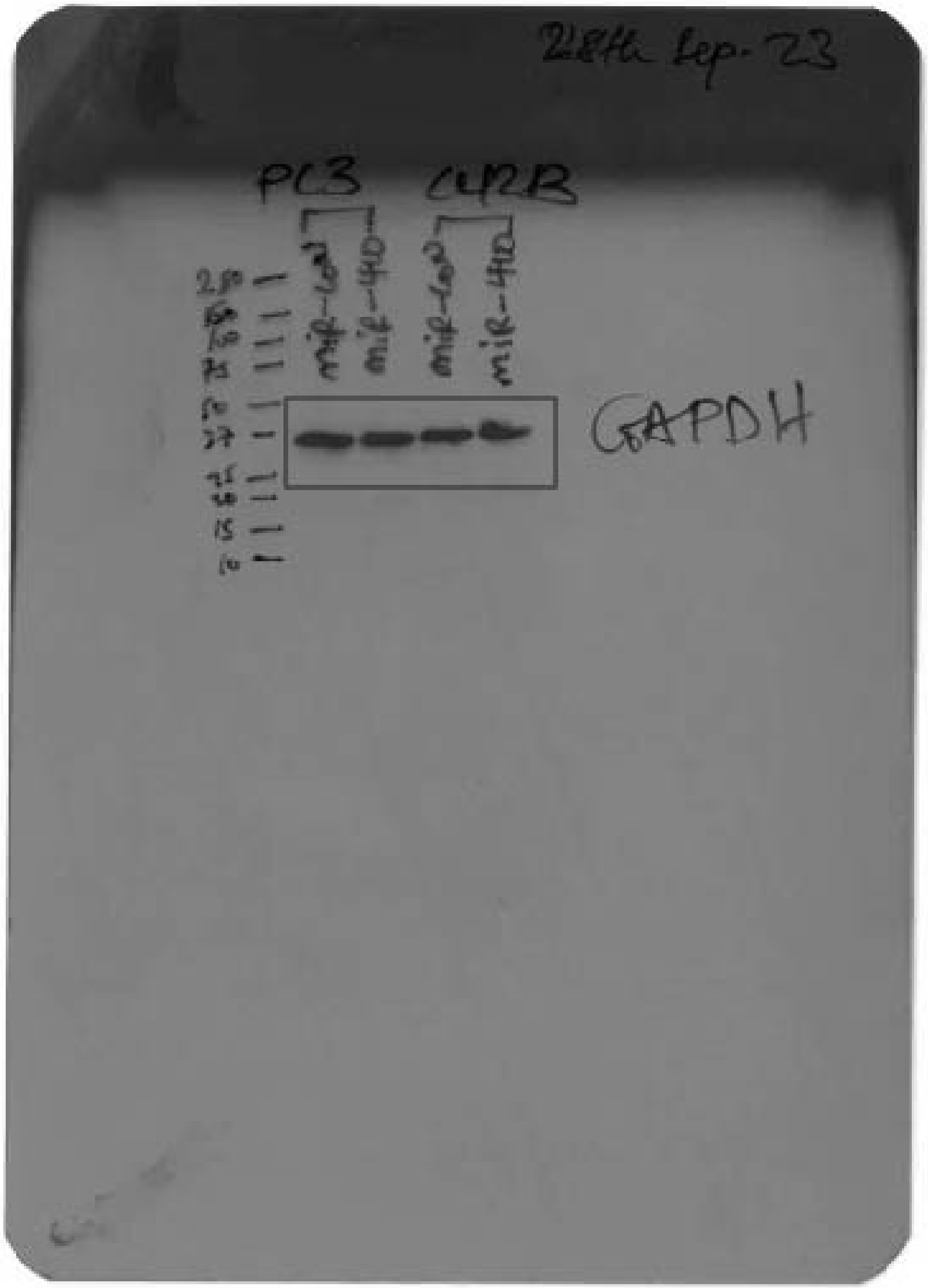

TGF-Beta blot for C42B miR-CON and C42B miR-410 as shown in Figure 7C

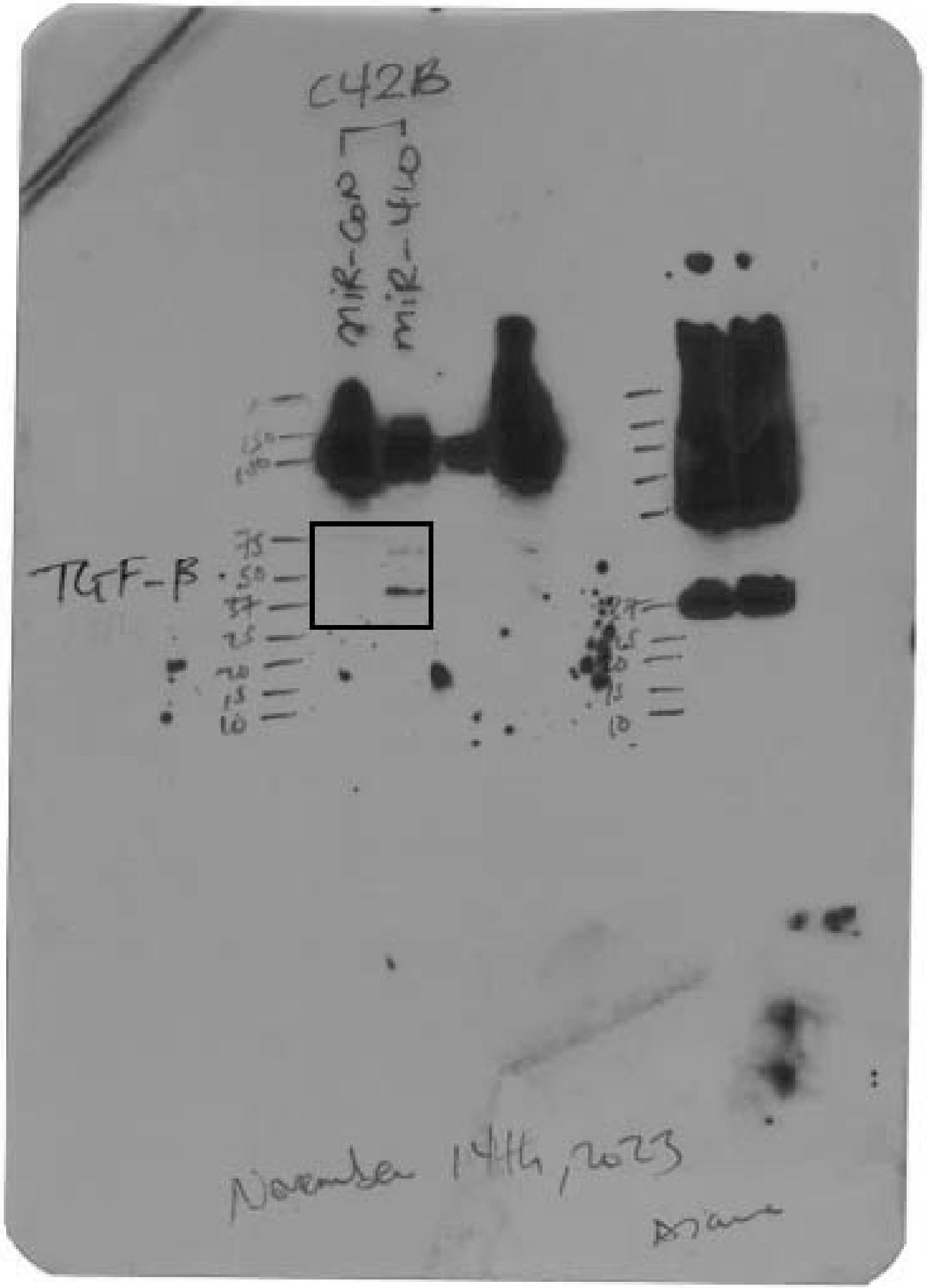

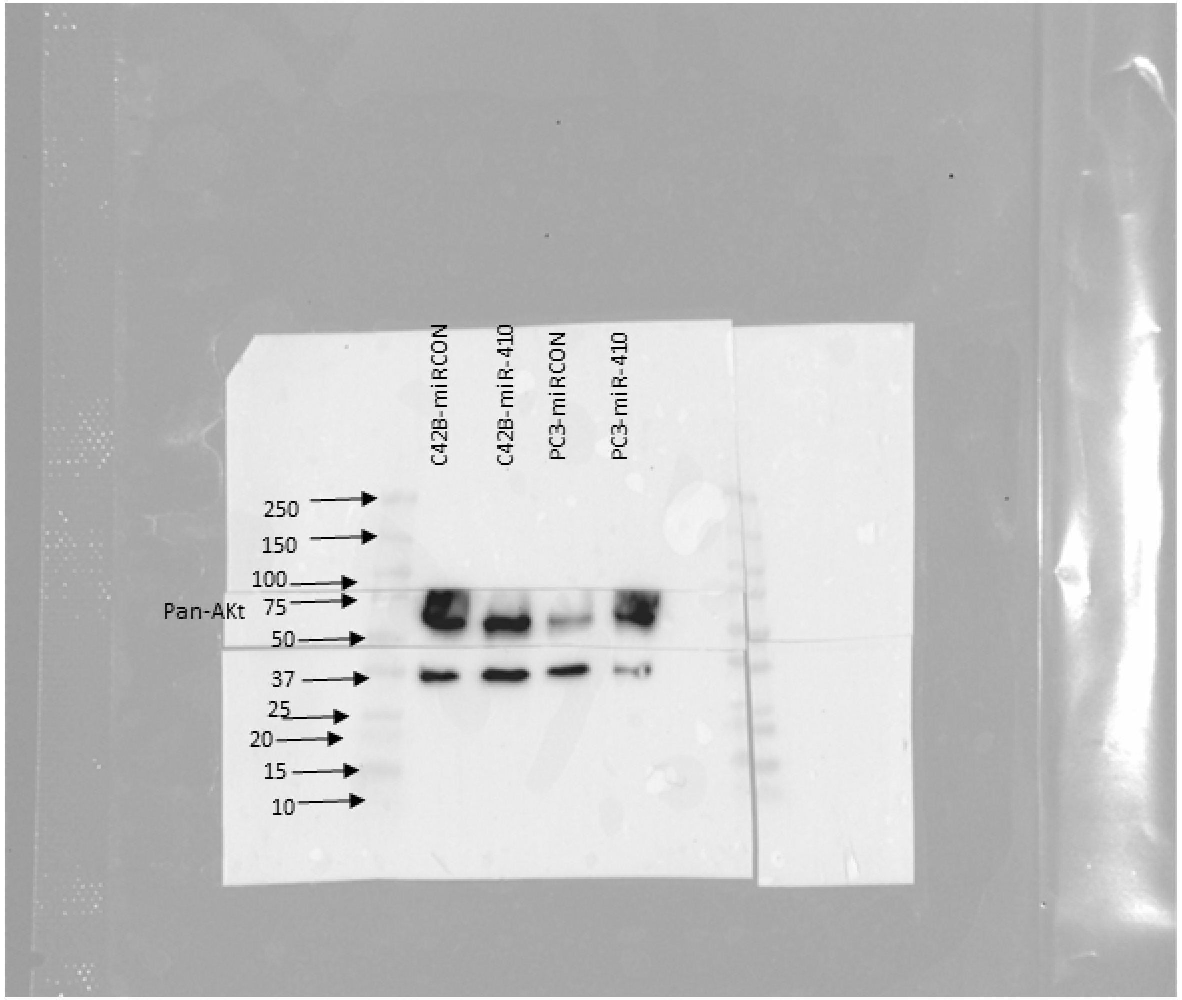

PanAkt for C42B miR-CON, C42B miR-410, PC3 miR-CON and PC3 miR-410 as shwon in Figure 7C
